# The More Educated, the Healthier: Evidence from Rural China

**DOI:** 10.3390/ijerph15122848

**Published:** 2018-12-13

**Authors:** Weidong Wang, Yongqing Dong, Xiaohong Liu, Linxiu Zhang, Yunli Bai, Spencer Hagist

**Affiliations:** 1 Center for Chinese Agricultural Policy, Institute of Geographic Sciences and Natural Resources Research, Chinese Academy of Sciences, Beijing 100101, China; wangwd.15b@igsnrr.ac.cn (W.W.); lxzhang.ccap@igsnrr.ac.cn (L.Z.); spencer.ccap@igsnrr.ac.cn (S.H.); 2 University of Chinese Academy of Sciences, Beijing 100101, China; 3 College of Management and Economics, Tianjin University, Tianjin 300072, China; dongyq.14b@igsnrr.ac.cn; 4 Center for Social Science Survey and Data, Tianjin University, Tianjin 300072, China; 5 China Center for Agricultural Policy, School of Advanced Agricultural Sciences, Peking University, Beijing 100871, China; xhliu2017@nsd.pku.edu.cn; 6 International Ecosystem Management Partnership, United Nations Environment Programme, Beijing 100101, China

**Keywords:** rural labor force, education, self-reported health, mental health, I2, I18, I21

## Abstract

Education, as an important aspect of human capital, not only affects the economic returns of an individual, but also affects non-economic returns. This paper uses data from the China Family Panel Studies (CFPS) in 2014 and explores the impact of education on the health status of rural residents by using the family fixed-effect model. We find that education can improve the self-reported health status and reduce the possibility of depression of rural residents. We also find that the effect of education on self-reported health status of rural young people more significant than that of middle-aged and old people, but the effect on depression score was weaker than that of middle-aged and old people. Compared with the high-income group, education improved the health of the lowest income group more significantly. Finally, we explore the mechanism of education affecting the health of rural residents from a multi-dimensional perspective.

## 1. Introduction

The economic returns of education have been widely confirmed by numerous researchers. For example, it can increase employment opportunities, raise income, and improve social and economic status, thus achieving upward mobility of careers [1,2,3,4,5]. However, the role of education is not limited to these. Recently, some studies have begun to focus on the non-economic returns of education and found that it is an important factor that can enhance people’s non-economic welfare [6,7].

Health as an important aspect of non-economic welfare, and the relationship between education and health has important policy implications. As the largest developing country, China’s economy has developed rapidly in the past four decades, and the urbanization rate reached 58.52% in 2017. However, China still has a large number of residents living in rural areas. In the meantime, as for the stock of human capital in the labor force in 2015, only 11.3 percent of adult workers in the 25–64 age bracket from rural areas had attained at least a high school education [8]. The health of rural residents is related to their livelihoods, the quality of China’s labor force, and the country’s future economic growth [9,10,11]. Based on this, we want to know: is improving the education level of rural residents an effective way to make them healthier in China?

Recent empirical studies have found a positive correlation between education and health. After adding some personal (such as age, gender, ethnicity, etc.) and family characteristics (such as family income, family demographic structure, etc.), the correlation remained significant [12,13,14,15,16,17,18]. However, whether the causal relationship between the two was correctly established ought to be tested; it is likely that this correlation is due to endogeneity [19,20,21].

Existing research has made a lot of headway in identifying the causal relationship between education and health, and has come to inconsistent conclusions. For example, Lleras-Muney [22] used the Compulsory Education Act of 1915–1939 in the United States as an instrumental variable to conclude that higher education levels can significantly reduce mortality. Berger [23] also used a dataset from America and found that the direct effect of schooling on health is more important than the effect of unobservables. Silles [24] adopted data from the General Household Survey for England, Scotland, and Wales and found that education has a positive impact on multiple health indicators, such as self-reported health, long-term illness, activity restrictions, and work restrictions. Other studies also support the above conclusions [25,26]. There have also been several studies which used the instrumental variable method and found that there is no evidence to indicate that education can improve people’s health, regardless of the health indicators chosen. For example, Jürges et al. [27] used the number of academic track schools in a state as an instrumental variable for years of schooling and investigated the causal effect of schooling on health behavior such as smoking and related outcomes such as obesity. They found large negative effects of education on smoking. Their study also indicated that there is no causal effect of education on reduced overweight and obesity. Arendt [28] found Education to be related to self-reported health (SRH), body mass index (BMI), and never having smoked in the expected ways, and that these relationships are amplified in magnitude when they use instrumental variables for education. However, as the standard errors also increase when using instruments, neither exogeneity nor the null of no effect of education can be rejected for SRH and BMI. Clark and Royer [29] used regression discontinuity methods and found little evidence that additional education improved health outcomes or changed health behaviors in Britain. Braakmann [30] drew almost the same conclusion to Clark and Royer’s study.

Though China is the most populous country in the world, it has attracted little attention in the literature. To our knowledge, there are only three studies which touch upon the causal effect of education on the health status of residents in China. Cheng et al. [31] found that education can improve the physical health and cognitive abilities of the elderly by using a sample from the Chinese Longitudinal Healthy Longevity Survey. In that study, they try to mitigate endogeneity problems by adding the initial health endowment variables and more control variables. Wang and He [32], based on the Propensity Score Matching method, found that tertiary education brought more health returns than high school education. Meanwhile, Xie and Mo [21] used China Health and Nutrition Survey (CHNS) data, using instrumental variable estimates, and found that greater education had no causal relationship with self-reported health, but can significantly reduce the probability of obesity in women.

These prior studies made a lot of useful attempts to explore the impact of education on the health of residents and lay a good foundation for our research. However, most of these studies have a limited contribution to policymaking because the causal relationships involved are ambiguous, and the earlier literature mainly focuses on data from developed countries and the topic of physical health, which leaves several open questions: does the causal effect of education on physical and mental health hold in developing countries such as China? Which group of people benefits most from educational attainment? Additionally, what is the underlying mechanism of education affecting health in rural China?

The overall goal of this paper is to extend this literature and provide more evidence for the causal effect of education on self-reported health and mental health in rural China by using the Family Fixed-Effects model. In line with this goal, we have three specific objectives. First, we seek to investigate whether there exists a causal relationship between the education and health status of rural residents. Second, we want to understand whether there exist any differences in this relationship across different age cohorts and income groups. Third, we will try to explore the possible mechanisms behind this relationship.

The paper unfolds as follows. Section 2 introduces the data we used and presents the descriptive statistical results. Section 3 and Section 4 describe the estimation strategy and the empirical results. Section 5 then discusses the possible mechanisms. Finally, Section 6 concludes.

## 2. Data and Descriptive Results

### 2.1. Data

This study uses the samples from China Family Panel Studies in 2014 (CFPS2014). CFPS is a nationally representative, annual longitudinal survey of Chinese communities, families, and individuals launched in 2010 by the Institute of Social Science Survey (ISSS) of Peking University in China. This survey is conducted every two years. However, since the data collected in 2016 has not released when we wrote the paper, therefore, the data we used was collected in 2014. The CFPS sample covers 25 provinces (including Provinces, Municipalities, and Autonomous Regions, not including the follow regions: Hong Kong, Macao, Taiwan, and Xinjiang Uygur Autonomous Region, Tibet Autonomous Region, Qinghai Province, Inner Mongolia Autonomous Region, Ningxia Hui Autonomous Region, and Hainan Province), and 162 counties and districts, which altogether represent 95% of China’s population. The target sample size is 16,000, and the survey targets all family members in the sample households. It mainly collects information on the economic and non-economic welfare of Chinese residents, as well as many research topics including health status, health behaviors, educational attainments, economic activities, family relationships and dynamic changes of the family, population migration, and subjective welfare. This dataset includes community, family and family relationship, adult, and children questionnaires. A total of 57,739 individuals were collected at the individual level. We only use rural samples who are 16 years or older and exclude those who were still in school from our study. Considering the missing values of some variables, the sample we used reaches 20,143 individuals.

### 2.2. Variables

#### 2.2.1. Definition of Variables

There are two main indicators to measure residents’ health. (1) Self-reported health, which is a physical health indicator. In the CFPS, respondents are asked “Right now, how do you describe your health status?”, after which they can choose only one answer, either “very poor”, “poor”, “fair”, “good”, or “excellent”. The value range is 1–5, for a total of 5 values. The higher the score, the healthier the individual is. (2) Depression tendency score (K6 Score). CFPS measures the mental health of residents by asking six questions about their mental health from within the last month. The six questions of K6 scale are as follows: First, “How often do you feel depressed, and unable to do anything exciting in the last month?” Second, “How often do you feel nervous?” Third, “Do you feel restless and have difficulty remaining calm?” Fourth, “How often do you feel hopeless about the future?” Fifth, “Do you feel difficulty doing anything?” Sixth, “How often do you think life is meaningless?” The subject being assessed has five options: never (0), sometimes (1), half (2), often (3), almost every day (4). These questions constitute the K6 scale developed by Kessler et al. [33] with support from the National Center for Health Statistics. The K6 scale has been widely shown to be an effective measure of mental health [34,35]. The score of the K6 scale is the sum of the scores of the six questions, ranging from 0 to 24. The higher the score, the worse the mental health status.

The key explanatory variable we focus on is the educational attainment of individual. CFPS collected the educational level of the individual, and the educational levels are divided into Illiterate/Semi-illiterate, Primary school, Junior high school, Senior high school, 2- or 3-year college, 4-year college/Bachelor’s degree, Master’s degree, and Doctorate degree. We also control for other variables, mainly including individual and family characteristics.

According to the existing literature, we also control for the individual and family characteristics of the respondents. Zhao [16] found that men’s health was better than women’s, and that as individuals grew older, their health became worse. Many studies also indicated that whether individuals had medical insurance also affected their health [36,37]. Therefore, we control for gender, age, and whether the individual has insurance. At the same time, individual who has a spouse can better share resources and risks. Therefore, we further add individual marital status to the model. Considering work affects the basic survival of individuals, we further add individual working conditions to the model. We also add the parents’ educational level, which also affects the quality of their childhood upbringing, into the model. As in Cheng [31], we also add the education level of individual parents as a control variable in the model. Family income affects access to medical resources and the quality of life of individuals, and further affects the health status of individuals [38]. Family size may also affect the allocation of family resources, thus affecting individual health. Judging from common sense, if we communicate with relatives and friends more frequently or the have a better relationship with neighbors, then the development of individual physical and mental health will be better. Therefore, we further control for the individual’s family income, family size (number of people living together), frequency of communication with relatives and friends (with the value ranging from 1 to 4; the greater the value, the more frequent the contact with relatives), and the relationship with neighbors (with the value ranging from 1 to 5; the greater the value, the better the relationship with neighbors), so as to further achieve a better estimate of the impact of education on health.

#### 2.2.2. Descriptive Statistics

Table 1 shows the descriptive statistics of variables. The mean score for the self-reported health of rural residents is 3.014 points, while the overall score for depression is 3.471 points. The educational level of rural residents is low. Males account for 51.2 percent of the sample, which reflects the demographic characteristics of rural China. Nearly 80 percent of our samples have spouses, 74.4 percent of them have jobs (including both agricultural and non-agricultural work), and nearly 90 percent have medical insurance. The average age is nearly 46 years old. The father’s education level is higher than mother’s, but neither reached the level of primary school graduation. The average annual net income of the family is 45,409 yuan.

To get a sense of the importance of educational attainment for individuals’ health status, we conduct a preliminary cross-tabulation analysis of these two factors. Cross-tabulation analysis is a data analysis method that simultaneously arranges two or more variables and their values in a statistical table in a certain order, so that the values of each variable become the nodes of different variables, from which the correlation between variables can be analyzed and a more scientific conclusion can be drawn. Table 2 reflects the relationship between education and the health of rural residents. We find that the better their self-reported health is, the better their mental health will be as individuals get better education. However, this is a preliminary judgement and the causality between the two needs to be further verified.

## 3. Empirical Strategy

In this paper, we adopt two methods to estimate the effect of educational attainment of individuals on their own health status. These two methods are the ordinary least squares (OLS) and family fixed effects, which are discussed below in detail.

### 3.1. The Ordinary Least Squares Model

As the benchmark estimation, we use the OLS model first. Here we should note that the theoretically appropriate specification of the model is an Ordered Logit or Ordered Probit equation, which captures the probability of respondents being in one category or the other more appropriately. However, with increasing usage of subjective well-being data and equations, researchers have found that the empirical results estimated from OLS equations are virtually identical to those from an Ordered Logit or Ordered Probit equations [39,40]. For convenience, we define $Edu_{i}$ as the education level of individual i. $Health_{i}$ represents the health status of individual i, which includes self-reported health and mental health. X represents other factors which may influence the individual’s health. We use regression Equation (1) to measure the effect of the education level on self-reported health and mental health:

(1) $$Health_{i}=\alpha_{i}+\beta edu_{i}+\delta X_{i}+u_{i}$$

β stands for the effect of individual’s educational attainment on their own health status. δ stands for the effect of other factors, and u is the error term assumed to be white noise.

### 3.2. Family Fixed-Effect Model

Considering that the unobservable factors of family inheritance, such as health characteristics, personal ability, and nurturing environment, affect both the educational attainment and health status of residents, this will bring about endogenous problems and ultimately leads to bias in the estimation results. Therefore, in order to further verify the robustness of the results, we use the family fixed-effect model (FFE) to try to alleviate endogeneity. The FFE, which has been used in many economic articles, is similar to the traditional fixed-effect model [41,42]. It constructs a panel-like data set, and then eliminates the factors that may affect both the education level and the health of individuals, which are unchanged over time, in the process of regression estimation.

Here we assume that members of the same family have similar genetic health traits and similar family parenting culture. The definitions of the explained variables are the same as with those in the OLS model. For each individual *i* in family *j*, we have:

(2) $$Health_{ij}=\alpha_{i}+\beta edu_{ij}+\delta X_{ij}+\mu_{j}+\varepsilon_{ij}$$

The meanings of the expressions are the same as above. $\mu_{j}$ is the unobservable characteristics shared in family *j*, and $\varepsilon_{ij}$ is the error term assumed to be white noise. A pooled regression is not appropriate since it ignores the unobservable characteristics $\mu_{i}$ shared in each family, which have an influence on both educational attainment and the health of the individual. Thus, we obtain the average at the family level as shown below:

(3) $$\bar{Health_{j}}=\bar{\alpha_{i}}+\beta \bar{edu_{j}}+\delta \bar{X_{j}}+\mu_{j}+\bar{\varepsilon_{j}}$$

By using the FFE model, we can eliminate $\mu_{j}$ from the equation by differentiating the above equation in the following way.

(4) $$Health_{ij}-\bar{Health_{j}}=\beta (edu_{ij}-\bar{edu_{j}})+\delta (X_{ij}-\bar{X_{j}})+\varepsilon_{ij}-\bar{\varepsilon_{j}}$$

## 4. Empirical Results

### 4.1. Ordinary Least Squares Results

Table 3 shows the results from OLS estimation using different measures of health. We find that the educational attainment of the individual has a significant effect on improving health status.

More specifically, a positive coefficient implies a positive covariation between schooling and better self-reported health. When we did not control for other variables, for each additional level of education increase, the score of self-reported health increased by 0.27, which is the simplest correlation we usually see between the two (row 1, column 1). When we put the variables of individual characteristics into the regression equation, the coefficient dropped to 0.055, which shows that health status is also affected by gender, age, and other factors, and that these personal characteristics dilute the impact of education on health (row 1, column 2). Taking into account some family characteristics may affect an individual’s own health status, so we further controlled for the family characteristics variables and the coefficient further dropped to 0.048 (row 1, column 3). Considering that some characteristics of villages, such as village infrastructure, drinking water, and sanitation, probably affect individual health status, we also controlled for village fixed effects to estimate the impact of education on health more effectively and the results are still robust (row 1, column 4).

The results of the correlation between education and depression indicate that educational attainment is significantly negatively correlated with the depression score of residents. When no control variables were added, with the increase of education level, the score of depression decreased by 0.563 (row 1, column 5). When we gradually controlled for individual and family characteristics and the village dummy variables, the coefficient decreased to 0.410, 0.339, and 0.254, respectively (row 1, columns 6 to 8). Considering that 30.9 percent of the rural residents’ depression scores were 0, we considered further using the Tobit model to verify the robustness of the estimates. The results show that education still plays an important role in the improvement of mental health (row 1, column 9).

### 4.2. Family Fixed-Effect Results

The robustness of the results verified in Table 4 is examined using FFE approach. When the individual characteristics variables are not included, the results obtained by using the family fixed-effect model are similar to those of the OLS estimation. However, the coefficient here is closer to 0.3, which is slightly higher than that of the OLS estimation (row 1, column 1). When individual characteristics were included, we find that the impact of education on self-reported health is still significant, and value decreased to 0.037 (row 1, column 2). The estimated results of the family fixed-effect model again confirm that the improvement of the education level can significantly reduce the depression score of residents (row 1, columns 3 to 5).

### 4.3. Heterogeneity Analysis

#### 4.3.1. Heterogeneity Analysis among Different Age Cohorts

We have come to the conclusion that education affects rural residents’ self-reported health and depression score. Yet as far as the timeframe is concerned, are there any differences between the short- and long-term effects of education? To explore this question, we divided our sample into two groups: the youth cohort (16 to 45 years old), and the middle-aged and older cohort (over 45 years old).

As shown in Table 5, we conclude that education has a significant positive impact on both short- and long-term self-reported health. For individuals aged 16 to 45 years old, self-reported health increases 0.058 levels for each additional level of education, while for those in the middle-aged and older cohort, the coefficient is smaller than that of the young cohort, at 0.031 (row 1, columns 1 and 2).

As for the results shown in columns 3 and 4, the effect of education on the depression score of residents was also different between the two age cohorts. For those in the young cohort, the coefficient on education is significant at the 1 percent level, which demonstrates that one level of education actually decreases the depression score by 0.227 points (row 1, column 3). For those aged above 45, the coefficient increased by 0.284 for each additional level of education, which was higher than that of the young cohort (row 1, column 4).

#### 4.3.2. Heterogeneity Analysis of Different Income Groups

Many studies have shown that increases in income can lead to health improvement [43,44]. Here, we want to know if the impact of education on health is different within different income groups. As such, we divided the individuals into four groups according to the per capita net income of the family. The empirical analysis showed that education had a positive and significant impact on the self-reported health and mental health of each income group. However, the role of education varies among different income groups (see Table 6).

Education has the greatest impact on the self-reported health of the lowest-income group. For each additional level of education, the self-reported health of the lowest-income group increased by 0.088 levels, while the coefficient is less than 0.05 for other income groups (row 1, columns 1 to 4). The same conclusion is also applicable to the influence of education on the mental health of residents in different income groups. Similarly, an increase in education had the greatest impact on the mental health of the lowest-income group. More specifically, each additional level of education lowered the depression score of rural residents in the lowest-income group by 0.349, compared to 0.155 in the highest-income group (row 1, columns 5 to 8).

## 5. Mechanism Analysis

What are the main channels for the impact of education on the health of residents? Grossman [45] pointed out that education has an impact on health primarily through two pathways. On the one hand, individuals with higher levels of education are better able to use healthcare resources to keep healthy. On the other hand, individuals with higher levels of education are able to maintain good habits and avoid behaviors that are detrimental to their health. There have also been many studies that try to conduct research along these dimensions [31,46,47]. 

We also believe that people with relatively lower education levels are more likely to have lower family professional statuses, poorer dietary conditions, poorer exercise habits, and less access to healthcare. As such, they may find more difficulty integrating into society, have worse standards of living, and face greater social pressures, so this may usher them into worse mental states. Considering the availability of data, the mechanism analysis is mainly prepared to carry out verification from the following three aspects in this paper. First of all, for rural Chinese residents, can a higher education level bring about the adoption of a healthy lifestyle? Second, can an increase in the education level raise income? Last, is an improvement in the education level able to improve subjective well-being? We believe that education can further affect the self-reported health and mental health of residents through these intermediate channels. 

### 5.1. Can Education Bring a Healthy Lifestyle to Individuals?

Here we focus on three common health behavior indicators: smoking, alcoholism, and exercise. We further explore the impact of education on health behaviors. First of all, we need to be clear on the indicators. The smoking indicator is a measure of whether the respondent smoked in the past month. The alcoholism index designates whether the respondent drank more than three times a week in the past month. The exercise indicator shows whether the respondent exercised in the past week.

As shown in Table 7, we can conclude that with the improvement of the education level, the probability of residents smoking and drinking is significantly reduced, while the possibility of exercise is increased (row 1, columns 1 to 3). This also attests that, in rural China, there is indeed a phenomenon in which education leads to a healthy lifestyle. Our findings are also consistent with the conclusions of Cheng et al. [31].

### 5.2. Can Education Increase Rural Residents’ Income?

We believe that there may be a mechanism for the impact of education on health. That is to say, the improvement of the education level of residents can bring about an improvement in income, thereby alleviating the budget constraints of residents and further affecting a series of health behaviors, such as increasing healthcare expenditures. In addition, the relative income status of residents can reflect their social status to some extent. The improvement of the education level of residents can bring about the increase of income and their relative income level, thus reducing their relative economic deprivation and improving their mental health.

To this end, we have selected two indicators of income status: one is the total income of individuals; the other is the relative income level. Among them, the relative income level refers to the rank of their income in their community, with 1 being the lowest and 5 being the highest, as scored by the respondents.

Table 8 shows that an improvement in the level of education can not only increase the individual’s total income, but also increase the relative income level. Specifically, for each level of education, the total personal income of residents increased by 1593.9 yuan, and the relative income level increased by 0.016.

### 5.3. Can Education Improve the Subjective Well-Being of Residents?

It is also possible to improve the mental health status of individuals and reduce their depression score by improving their subjective welfare. We select the respondents’ satisfaction with their own lives, their satisfaction with their marital lives, and their confidence in their future to measure subjective well-being. Here, the three subjective welfare measures are set to three 0–1 dummy variables.

We conclude that education has a significant positive impact on the improvement of subjective well-being (Table 9). That is to say, with the improvement of the education level, individuals improved their life satisfaction, marital satisfaction, and confidence in their future. Specifically, for each additional level of education, the likelihood of rural residents being satisfied with their own lives increased by 1.1%, while the likelihood of satisfaction with marriage also increased by 0.1%, and the likelihood of being confident in their future increased by nearly 2% (row 1, columns 1 to 3). As such, we believe that education could promote the improvement of the subjective welfare of rural residents, and thereby improve their mental health.

## 6. Conclusions

This study uses the China Family Panel Studies in 2014 to explore the impact of education on the health of rural residents. The results show that the improvement of the education level can significantly improve the self-reported health and mental health status of rural residents in China. We further find that the impact of education on the health of rural people is heterogeneous. That is, education has a bigger effect on the improvement of self-reported health of young people than of middle-aged and older people in rural areas, and the effect of education on depression is slightly weaker for middle-aged and older people. In addition, the impact of education on rural people from families of differing incomes is also non-uniform. Specifically, the effect of education is more pronounced for those individuals from low-income families. Further exploration of the mechanisms show that education improves health by improving individuals’ healthy living habits, loosening budget constraints, and improving subjective well-being.

The results of this study have relatively strong policy implications. First, the contribution of education to human capital investment in rural areas is not only reflected in income growth, but also in the improvement of individuals’ health, which means that education is regarded as an important non-economic value of human capital. At the same time, education and health are both important human capital components. Improving education can improve healthy human capital, and it also provides some reference for projects or national strategies that aim to improve health. 

Second, education’s impact on the improvement of health is a continuous process, and is not only manifested in short-term effects. The impact of education on the health of middle-aged and elderly people is also considerable, especially the obviously improvement of mental health in rural middle-aged and elderly groups. 

Third, the conclusion that the poorest benefit most from the improvement of educational attainment, which may lead us to ponder over the current poverty alleviation strategies in developing countries. Developing countries, especially China, are striving to achieve poverty alleviation. The government is also advocating for poverty alleviation through education. According to our study, investing in education is an effective strategy for conducting poverty alleviation, and can bring more returns while effectively avoiding the phenomenon of sliding back into poverty.

Cheng et al. [48] believed that, from the perspective of narrowing the income gap, and in consideration of education and health, the policy orientation of poverty reduction should be to prioritize the improvement of residents’ health. However, our study indicates that education and health are inextricable components within the framework of poverty alleviation.

Our study faces a series of potential pitfalls. First, although this study attempts to alleviate the endogeneity problem through the family fixed-effect model, the assumptions we made are still too strong. In addition, mental health also includes other dimensions besides depression we focus on.

## Figures and Tables

**Table 1:** Descriptive statistics of variables.

Variables	N (1)	Mean (2)	S.D. (3)	Min (4)	Max (5)
***Dependent variables***					
Self-reported health	20,143	3.014	1.303	1	5
Depression score	16,421	3.471	4.154	0	24
***Key explanatory variable***					
Educational level	20,143	2.248	1.194	1	7
***Individual characteristics***					
Male (1 = Yes, 0 = No)	20,143	0.512	0.500	0	1
Have a spouse (1 = Yes, 0 = No)	20,142	0.798	0.401	0	1
Has a job (1 = Yes, 0 = No)	17,666	0.744	0.436	0	1
Age	20,143	45.949	16.794	16	104
Medical insurance	20,082	0.899	0.302	0	1
Father’s educational level	15,559	1.802	0.980	1	8
Mother’s educational level	15,727	1.358	0.707	1	8
***Family Characteristics***					
Net household income	18,556	45,409.320	39,888.170	1	270,500
The number of people living together in a family	19,999	4.623	2.049	1	17
Frequency of communication with relatives and friends	19,616	1.396	0.753	0	3
Relationship between family members and neighbors	19,615	1.860	0.855	1	5

Data source: China Family Panel Studies 2014.

**Table 2:** Cross analysis of education and health, (%).

Health Status	Illiterate/Semi-Illiterate	Primary School	Junior High School	Senior High School	2 or 3 Years College	4-Year College/Bachelor’s Degree	Master’s Degree or above
***Self-reported health***							
1	31.1	16.3	9.0	8.3	2.4	1.4	0.0
2	15.9	15.5	12.9	12.3	9.0	7.8	0.0
3	26.8	30.9	33.9	36.2	35.2	35.7	26.7
4	16.0	21.3	25.0	25.5	28.4	32.7	60.0
5	10.2	16.1	19.2	17.8	25.0	22.5	13.3
Sum	100	100	100	100	100	100	100
***Depression score***							
0–4	62.2	71.8	78.1	78.6	81.4	76.9	91.7
5–9	23.5	19.3	16.4	16.8	15.7	18.9	8.3
10–14	9.8	6.5	4.2	3.4	1.7	3.6	0.0
15–19	3.3	2.1	1.1	1.1	1.2	0.6	0.0
20–24	1.2	0.4	0.3	0.1	0.0	0.0	0.0
Sum	100	100	100	100	100	100	100

Notes: (i) Data source: China Family Panel Studies 2014. (ii) Since the depression score (K6 Score) is a continuous variable with many values, if we make a cross-tabulation analysis between the individual’s education and all mental status scores, it will present a very large table. Therefore, in order to facilitate ease of use for the reader, we divide the depression score into several stages. The detailed distribution of the depression score is shown in Appendix A, Table A1.

**Table 3:** The impact of education on health (OLS).

	Self-Reported Health				Depression Score				
Independent Variables	(1)	(2)	(3)	(4)	(5)	(6)	(7)	(8)	Tobit (9)
Educational	0.270 ***	0.055 ***	0.048 ***	0.051 ***	−0.563 ***	−0.410 ***	−0.339 ***	−0.254 ***	−0.137 ***
level	(0.007)	(0.010)	(0.010)	(0.011)	(0.026)	(0.035)	(0.036)	(0.037)	(0.028)
Individual characteristics	N	Y	Y	Y	N	Y	Y	Y	Y
Family characteristics	N	N	Y	Y	N	N	Y	Y	Y
Village fixed effect	N	N	N	Y	N	N	N	Y	Y
R-squared	0.061	0.151	0.165	0.247	0.025	0.037	0.050	0.181	-
Observations	20,143	12,590	11,982	11,805	16,421	11,995	11,362	11,188	11,188

Notes: (i) Data source: China Family Panel Studies 2010. (ii) Robust standard errors in parentheses. (iii) ***, **, and * indicate statistical significance from zero at the 1, 5, and 10 percent levels. (iv) We also used Order Probit model to identify the impact of education on self-reported health and draw a similar conclusion. (V) Column 9 reports the marginal effect of education on depression score.

**Table 4:** The impact of education on health (FFE).

	Self-Reported Health		Depression Score		
Independent Variables	(1)	(2)	(3)	(4)	Xttobit (5)
Educational level	0.297 ***	0.037 **	−0.408 ***	−0.111 **	−0.193 ***
	(0.009)	(0.015)	(0.034)	(0.050)	(0.024)
Individual characteristics	N	Y	N	Y	Y
R-squared	0.071	0.165	0.015	0.036	-
Observations	20,143	12,950	16,421	11,995	11,995
Number of families	7273	6449	6936	6144	6144

Notes: (i) Data source: China Family Panel Studies 2010. (ii) Robust standard errors in parentheses. (iii) ***, **, and * indicate statistical significance from zero at the 1, 5, and 10 percent levels. (iv) Column 5 reports the conditional marginal effect of education on depression score result.

**Table 5:** The impact of education on health by age cohorts (OLS).

	Self-Reported Health		Depression Score	
	16–45	Above 45	16–45	Above 45
Independent Variables	(1)	(2)	(3)	(4)
Educational level	0.058 ***	0.031 *	−0.227 ***	−0.284 ***
	(0.014)	(0.018)	(0.051)	(0.058)
Individual characteristics	Y	Y	Y	Y
Family characteristics	Y	Y	Y	Y
Village fixed effect	Y	Y	Y	Y
R-squared	0.209	0.188	0.198	0.224
Observations	6051	5754	5551	5637

Notes: (i) Data source: China Family Panel Studies 2010. (ii) Robust standard errors in parentheses. (iii) ***, **, and * indicate statistical significance from zero at the 1, 5, and 10 percent levels.

**Table 6:** The impact of education on health by age cohorts, by per capita net income of family.

	Self-Reported Health				Depression Score			
	Lowest	Lower	Higher	Highest	Lowest	Lower	Higher	Highest
Independent Variables	(1)	(2)	(3)	(4)	(5)	(6)	(7)	(8)
Educational level	0.088 ***	0.045 *	0.049 **	0.045 **	−0.349 ***	−0.312 ***	−0.269 ***	−0.155 **
	(0.026)	(0.024)	(0.023)	(0.020)	(0.096)	(0.083)	(0.073)	(0.067)
Individual characteristics	Y	Y	Y	Y	Y	Y	Y	Y
Family characteristics	Y	Y	Y	Y	Y	Y	Y	Y
Village fixed effect	Y	Y	Y	Y	Y	Y	Y	Y
R-squared	0.327	0.296	0.331	0.292	0.277	0.275	0.235	0.264
Observations	2962	2937	2965	2941	2821	2766	2821	2780

Notes: (i) Data source: China Family Panel Studies 2010. (ii) Robust standard errors in parentheses. (iii) ***, **, and * indicate statistical significance from zero at the 1, 5, and 10 percent levels. (iv) The different income groups are divided into four groups according to the net income per capita of the sample we used. The lowest income group is 0–3885 yuan per capita net income; the lower income group is 3885–8250 yuan per capita net income; the higher income group is per capita net income range from 8250 to 14,161.25 yuan; the highest income group is above 14,161.25 yuan.

**Table 7:** The impact of education on health behavior (Probit).

	Health Behavior		
Explanatory Variable	Smoking (1 = yes, 0 = no)	Alcoholism (1 = yes, 0 = no)	Exercise (1 = yes, 0 = no)
Educational level	−0.026 ***	−0.010 ***	0.055 ***
	(0.003)	(0.004)	(0.011)
Individual characteristics	Y	Y	Y
Family characteristics	Y	Y	Y
Village fixed effects	Y	Y	Y
observation	11,034	10,498	11,546

Notes: (i) Data source: China Family Panel Studies 2010. (ii) Robust standard errors in parentheses. (iii) ***, **, and * indicate statistical significance from zero at the 1, 5, and 10 percent levels. (iv) The coefficient in probit model is the standardized marginal effect.

**Table 8:** The impact of education on personal income (OLS).

	Income	
Explanatory Variable	Total Personal Income (Yuan)	Relative Income Level
Educational level	1593.931 ***	0.016 *
	(163.465)	(0.009)
Individual characteristics	Y	Y
Family characteristics	Y	Y
Village fixed effects	Y	Y
R-squared	0.275	0.113
Observation	11,798	11,385

Notes: (i) Data source: China Family Panel Studies 2010. (ii) Robust standard errors in parentheses. (iii) ***, **, and * indicate statistical significance from zero at the 1, 5, and 10 percent levels.

**Table 9:** The impact of education on subjective well-being (Probit).

	Subjective Well-Being		
Explanatory Variable	Satisfied with Own Lives (1 = yes, 0 = no)	Satisfaction with Marriage (1 = yes, 0 = no)	Confidence in Their Future (1 = yes, 0 = no)
Educational level	0.011 **	0.001 *	0.018 ***
	(0.010)	(0.010)	(0.010)
Individual characteristics	Y	Y	Y
Family characteristics	Y	Y	Y
Village fixed effects	Y	Y	Y
Observations	11,068	8725	11,001

Notes: (i) Data source: China Family Panel Studies 2010. (ii) Robust standard errors in parentheses. (iii) ***, **, and * indicate statistical significance from zero at the 1, 5, and 10 percent levels. (iv) The coefficient in probit model is the standardized marginal effect.

## Appendix A

**Table A1:** Cross analysis of education and depression score.

Depression Score	Illiterate/Semi-Literate	Primary School	Junior High School	Senior High School	2 or 3 Years College	Bachelor’s Degree	Master’s Degree	Total
0	1574	1407	1477	450	120	47	3	5078
1	555	526	608	229	63	20	2	2003
2	524	395	498	172	60	24	4	1677
3	552	372	419	136	48	20	0	1547
4	484	300	370	110	46	19	2	1331
5	382	254	230	74	20	12	0	972
6	391	236	220	83	28	14	1	973
7	229	132	99	36	10	1	0	507
8	233	97	85	29	2	3	0	449
9	161	86	72	13	5	2	0	339
10	147	78	46	22	3	4	0	300
11	116	58	48	10	1	2	0	235
12	154	47	40	9	3	0	0	253
13	93	48	26	4	0	0	0	171
14	71	39	21	2	0	0	0	133
15	58	23	8	4	3	0	0	96
16	52	14	13	4	2	0	0	85
17	20	18	12	4	0	1	0	55
18	44	18	9	2	0	0	0	73
19	24	14	5	1	0	0	0	44
20	16	10	5	0	0	0	0	31
21	12	2	2	1	0	0	0	17
22	8	3	1	0	0	0	0	12
23	12	0	0	0	0	0	0	12
24	21	3	3	1	0	0	0	28
Total	5933	4180	4317	1396	414	169	12	16,421

Data source: China Family Panel Studies 2014.
